# Recent insights into Wzy polymerases and lipopolysaccharide O-antigen biosynthesis

**DOI:** 10.1128/jb.00417-24

**Published:** 2025-03-11

**Authors:** Alice Ascari, Renato Morona

**Affiliations:** 1 Institute for Biomedicine and Glycomics, Griffith University 97562 , Gold Coast, Queensland, Australia; 2 School of Biological Sciences, Department of Molecular and Biomedical Science, Research Centre for Infectious Diseases, University of Adelaide 110455 , Adelaide, Australia; University of Notre Dame, Notre Dame, Indiana, USA

**Keywords:** lipopolysaccharide, O-antigen, Wzy, polymerases, Wzz, co-polymerases, polysaccharides, Wzy-dependent pathway

## Abstract

Bacteria synthesize a plethora of complex surface-associated polysaccharides which enable them to persist and thrive in distinct niches. These glycans serve an array of purposes pertaining to virulence, colonization, antimicrobial resistance, stealth, and biofilm formation. The Wzx/Wzy-dependent pathway is universally the predominant system for bacterial polysaccharide synthesis. This system is responsible for the production of lipopolysaccharide (LPS) O-antigen (Oag), enterobacterial common antigen, capsule, and exopolysaccharides, with orthologs present in both Gram-negative and Gram-positive microbes. Studies focusing principally on *Pseudomonas*, *Shigella*, and *Salmonella* LPS Oag synthesis have provided much of the framework underpinning the biochemical and molecular mechanism behind polysaccharide synthesis via this pathway. LPS Oag production via the Wzx/Wzy-dependent pathway occurs through the stepwise activity of multiple key biosynthetic enzymes, including primarily the polymerase, Wzy, which is responsible for the Oag assembly, and the polysaccharide co-polymerase, Wzz, which effectively modulates the length of the glycan produced. In this review, we provide a comprehensive summary of the latest genetic, structural, and mechanistic data for the main protein candidates of the Wzx/Wzy-dependent pathway, in addition to an examination of their substrate specificities. Furthermore, we have reviewed recent insights pertaining to the dynamics/kinetics of glycan synthesis by this mechanism, including the interplay of the key proteins among themselves and in complex with their substrate. Lastly, we outline key gaps in the literature and suggest future research avenues, with the aim to stimulate ongoing research into this critical pathway responsible for the production of key virulence factors for numerous debilitating and lethal pathogens.

## INTRODUCTION

Bacteria have evolved to synthesize a plethora of complex surface-associated glycans that enable them to persist and thrive in distinct niches (1, 2). These surface polysaccharides (SPs), particularly capsule, lipopolysaccharide (LPS) with an O-antigen (Oag) domain, enterobacterial common antigen (ECA), and exopolysaccharide, are known to evolve rapidly and play a central role in bacterial classification (3–5). Collectively, SPs serve a multitude of purposes, pertaining to enhancing virulence, facilitating colonization, conferring resistance to a variety of antimicrobials, and aiding in stealth and biofilm formation (6–11). Different bacterial species and strains exhibit distinct SP populations that protect them and equip them for their interactions with their specific extracellular milieu (1).

**Fig 1:**
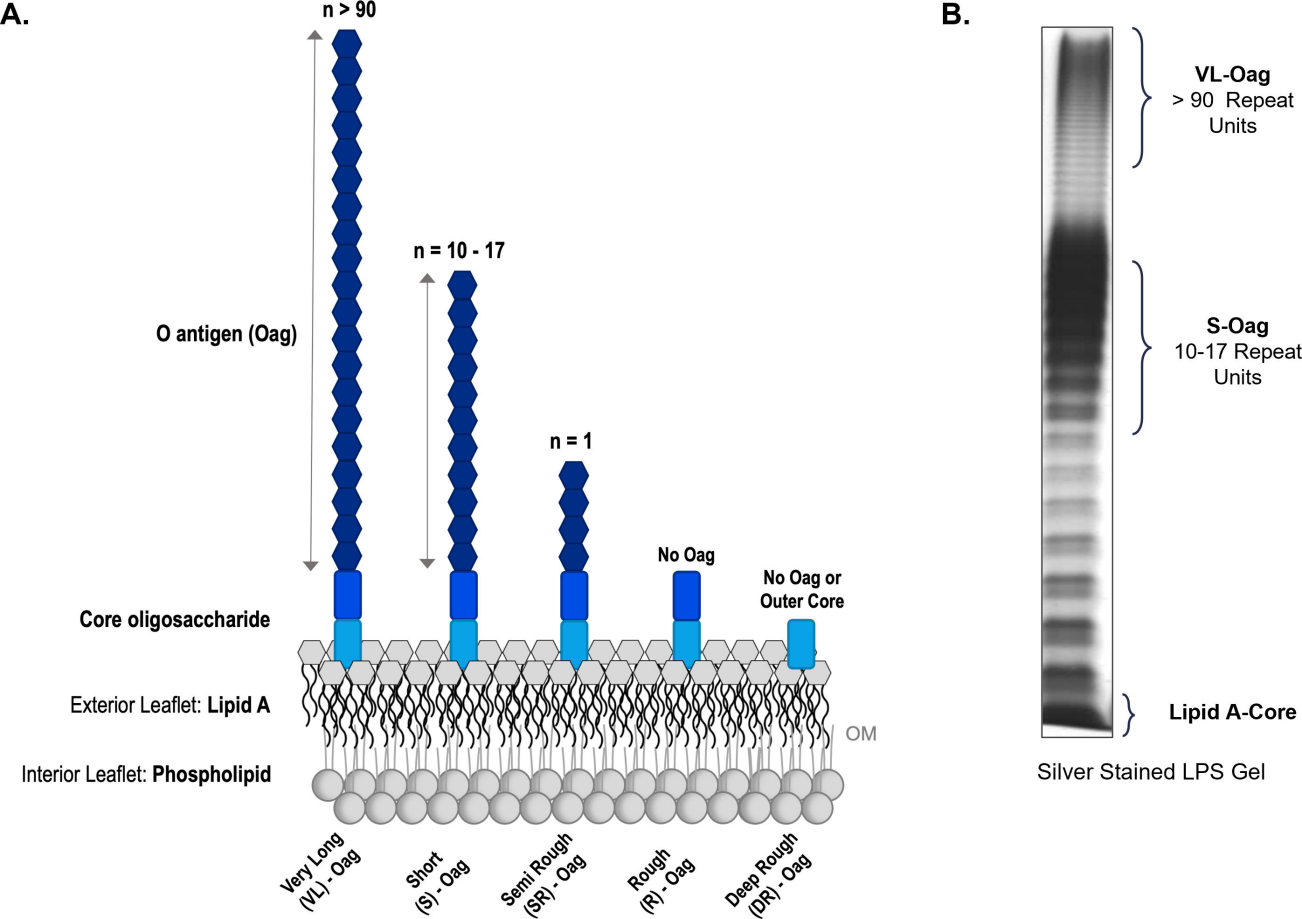
The structure of LPS and mutant phenotypes. (A) An overview of wild-type and mutant LPS presentations. *Shigella flexneri* LPS is a glycolipid expressed on the exterior leaflet of the bacterial outer membrane (OM). LPS can be subdivided into three distinct domains: (i) lipid A, which forms the exterior leaflet of the OM bilayer; (ii) a core oligosaccharide; and (iii) a highly variable Oag polysaccharide. From left to right: very long Oag (VL-Oag) LPS, a wild-type LPS molecule with an Oag component containing over 90 tetrasaccharide RUs; short Oag (S-Oag) LPS, another wild-type LPS molecule, instead containing an Oag domain with 10–17 RUs; SR-Oag LPS, a mutant LPS presentation, whereby molecules contain only a single Oag RU; R-Oag LPS, another mutant LPS phenotype, in which LPS molecules are entirely devoid of the Oag component; and finally DR-Oag, a mutant LPS molecule devoid of both the Oag and the outer residues of the oligosaccharide component. (B) Silver-stained analysis of *S. flexneri* serotype Y, expressing VL-Oag and S-Oag. The distinct LPS Oag presentations can be differentiated based on the distribution of their molecular masses.

*Enterobacteriaceae*, in particular, are characterized by the expression of a heterogeneous population of two primary surface molecules: LPS and ECA (8, 12). The expression of both glycolipids on the OM surface protects bacteria from assault by niche antimicrobial agents, such as bile salts and dietary lipids, which are characteristics of the digestive tract through which they pass to reach and infect their colonization niche (2, 10, 13, 14). Namely, LPS also contributes significantly to bacterial virulence, serving as a key adhesion molecule, a major structural component of the OM, and its amphiphilic nature also further modulates assault from host immunoregulatory responses, such as complement (3, 9, 11, 15, 16). LPS can be structurally subdivided into three distinct domains: (i) lipid A at the proximal end, which serves as the anchor for LPS molecules in the OM; (ii) an oligosaccharide core, which can be further subdivided into inner and outer sugars; and (iii) the Oag polysaccharide at the distal end (Fig. 1A) (3, 17–20).

### O-antigen

The distal domain of LPS, the Oag polysaccharide, is a highly variable, long glycan polymer consisting of oligosaccharide repeat units (RUs) (3). The composition of Oag RUs characteristically contains three to six sugar moieties; however, the identity and arrangement of these composite sugar residues vary widely across distinct bacterial species/serotypes (8, 21). Notably, some microbes incorporate charged sugar residues in their LPS Oag, which in turn significantly influence their surface chemistry. For instance, *Pseudomonas aeruginosa* incorporates uronic acid in its trisaccharide Oag RUs, leading to the synthesis of a negatively charged sugar polymer (22, 23). The high Oag heterogeneity observed across Gram negatives also arises due to the incorporation of different stereochemistry (α- and β-linkages) between sugar residues and the modification of RUs with chemical substituents (such as acetyl or methyl groups) and/or entire sugar moieties (typically fucosyl or glucosyl groups) (8, 24–26).

LPS can be described as either smooth (S-) or rough (R-) based on its O-antigenic presentation, where S-LPS molecules carry a full-length Oag polysaccharide and R-LPS are instead completely devoid of this domain (16, 27). Furthermore, some pathogens, such as *Shigella flexneri*, can express a heterogeneous population of two distinct S-LPS forms unique in their Oag presentation, referred to as short (S-Oag) and very long (VL-Oag) Oag LPS (Fig. 1A and B) (28–30). Specifically, these LPS Oag molecules vary in their length, with the former consisting of 10–17 RUs and the latter containing over 90 tetrasaccharide repeats (Fig. 1A and B) (31). Studies performed in multiple bacterial models have additionally demonstrated that mutagenesis of key LPS biosynthetic enzymes, which disrupt glycolipid production and often cause the accumulation of lipid-linked glycan intermediates (2, 32), culminates in novel Oag phenotypes (summarized in Fig. 1A). These presentations include deep-rough Oag (DR-Oag), which refers to LPS molecules devoid of both the Oag polysaccharide and the outer residues of the core oligosaccharide; rough Oag LPS (R-Oag), which describes LPS molecules entirely and exclusively devoid of the Oag domain; or semi-rough Oag LPS (SR-Oag), which sees the ligation of only a single Oag RU to the lipid A-core (27, 33).

### LPS Oag synthesis: the Wzx/Wzy-dependent pathway

In Gram negatives, multiple pathways exist for synthesizing OM polysaccharides. These systems include the ABC transporter-dependent pathway, the synthase-dependent pathway, and the Wzx/Wzy-dependent pathway (8, 34, 35). The Wzx/Wzy-dependent pathway is the predominant system for polysaccharide synthesis in bacteria. This mechanism is responsible for the synthesis of LPS Oag, ECA, capsule, and exopolysaccharides, with orthologs of the pathway present in Gram-negative and Gram-positive microbes (3, 8). Studies involving *Escherichia coli*, *S. flexneri*, *P. aeruginosa*, *Salmonella* spp., and *Yersinia* spp. have provided much of the framework underpinning the biochemical and molecular mechanisms behind polysaccharide synthesis via this pathway (8, 28, 29, 35–39).

**Fig 2:**
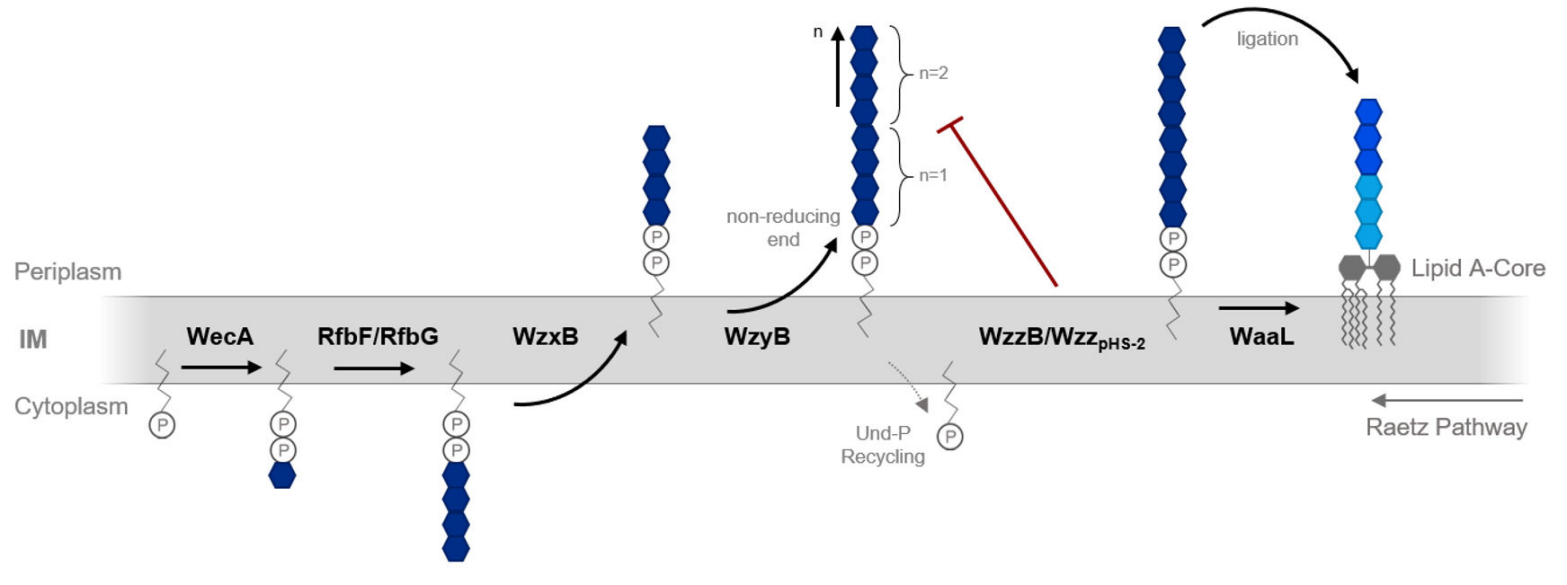
*Flexneri* LPS Oag biosynthesis via the Wzx/Wzy-dependent pathway. LPS Oag biosynthesis begins on the cytoplasmic surface of the IM, whereby the glycosyltransferase WecA and rhamnosyltransferases RfbF and RfbG sequentially add a single GlcNAc and three Rha moieties to the membrane-associated carrier lipid Und-P. Upon the assembly of the full Und-PP-linked tetrasaccharide, the complete Oag RU is translocated across the IM by the flippase WzxB and captured by the polymerase WzyB. WzyB adds the nascent Oag RU to the nonreducing end of the growing Oag polymer. Termination of Oag elongation is regulated by two competing polysaccharide co-polymerase proteins, WzzB and Wzz_pHS-2_, to mediate the synthesis of the S-Oag and VL-Oag polysaccharides, respectively. The complete Oag is then ligated to a lipid A-core oligosaccharide molecule by the catalytic activity of WaaL, prior to export of the full LPS molecule to the OM putatively mediated by the Lpt system (not shown).

Pathogens, such as *S. flexneri*, implement the Wzx/Wzy-dependent pathway to synthesize LPS Oag. In brief, polysaccharide production commences with the assembly of Oag tetrasaccharide RUs, consisting of a single *N*-acetylglucosamine (GlcNAc) and three successive rhamnose (Rha) residues, at the cytoplasmic leaflet of the inner membrane (IM) (Fig. 2) (37). WecA, a membrane-bound glycosyltransferase (GT), utilizes the donor UDP-GlcNAc to first transfer a single GlcNAc moiety to undecaprenol phosphate (Und-P) in an Mg^2+^/Mn^2+^-dependent process. Subsequently, rhamnosyltransferases RfbG and RfbF then complete the full undecaprenol diphosphate-linked Oag RU (Und-PP-RU) through the stepwise ligation of the three Rha residues, using the precursor dTDP-Rha (Fig. 2) (37, 40–42). Once assembled, the Und-PP-RU is next translocated to the periplasmic face of the IM by the polysaccharide transport protein, or “flippase,” WzxB_SF_, before its transfer to the polymerase, WzyB_SF_ (6, 8, 37, 43). Notably, while WzxB proteins were initially believed to recognize and bind the undecaprenol diphosphate moiety of Und-PP-RUs (44, 45), subsequent studies demonstrated that these translocation proteins also exhibit a high degree of specificity to both the first saccharide and the overall chemical structure of the RU (46). WzyB_SF_ is then responsible for the repeat addition of these RUs to the nonreducing end of the growing polysaccharide chain in an energy-intensive manner (43). Importantly, the termination of the Oag synthesis is regulated by two competing polysaccharide co-polymerases, otherwise termed the chain length determinants (Fig. 2) (47). The first, WzzB_SF_, is encoded on the bacterial chromosome and modulates the synthesis of S-Oag LPS (37, 48). The latter, Wzz_pHS-2_, is instead encoded on a small plasmid (pHS-2; 3 kb) and regulates the production of the VL-Oag modal length (31, 49). In some pathogens, such as *Yersinia pseudotuberculosis* O:2 a, the presence of the Wzz polysaccharide co-polymerase is essential for wild-type LPS Oag synthesis (50). Once polymerized, the complete Oag chain is linked to the lipid A–core oligosaccharide component through the activity of the WaaL polysaccharide ligase (Fig. 2) (43, 48). Finally, the export of the full LPS molecule to the OM is likely mediated by the Lpt system (51). This proposed mechanism involves the activity of seven key integral and soluble proteins, which together work in complex to excise, transport, and correctly insert the mature LPS molecule into the OM (51–55).

### Wzy polymerases

Wzy proteins belong to the shape, elongation, division, and sporulation (SEDS) protein family, a broad class of highly hydrophobic, polytopic IM proteins involved in cell envelope biosynthesis (56). Since their first detection, Wzy polymerases have been shown to exert anabolic function, specifically through the catalysis of new glycosidic linkages between nascent, single Oag RUs to the reducing terminus of a growing sugar polymer (composed of multiple RUs of the same Oag oligosaccharides) (3, 57, 58). Thus, Wzy polymerases are widely classified as a unique family of GTs, which implement beta-fold GT activity due to their mechanism of glycosidic bond formation (i.e., no requirement for divalent cations) (57, 59), specifically their ability to concurrently accept a donor substrate (nascent RU) while retaining an acceptor substrate (growing polysaccharide) (23, 36, 59) and their use of a donor substrate linked to Und-PP rather than a conventional nucleotide-phosphate sugar (60). The literature has implemented Wzy proteins in the synthesis of an array of complex polysaccharides, including LPS Oag, capsule, ECA, and exopolysaccharide (8).

### Wzy identification and genetic organization

The genes encoding the LPS Oag biosynthetic machinery were originally characterized in *Salmonella* spp. and *E. coli* K-12 via genetic linkage analyses, employing bacterial conjugation and bacteriophage transduction experiments (61–63). These pioneering studies led to the identification of the *rfb* locus, which, in combination with a series of subsequent genetic and biochemical investigations spanning over 40 years, was shown to contain all the genes necessary for LPS Oag synthesis (43, 57, 64–66). Notably, despite its early identification, it was not until the *in vitro* reconstitution of the Wzy-dependent pathway by Woodward and colleagues (57) that *wzy* (previously known as *rfc*) was unequivocally confirmed to encode the Oag polymerase (57).

**Fig 3:**
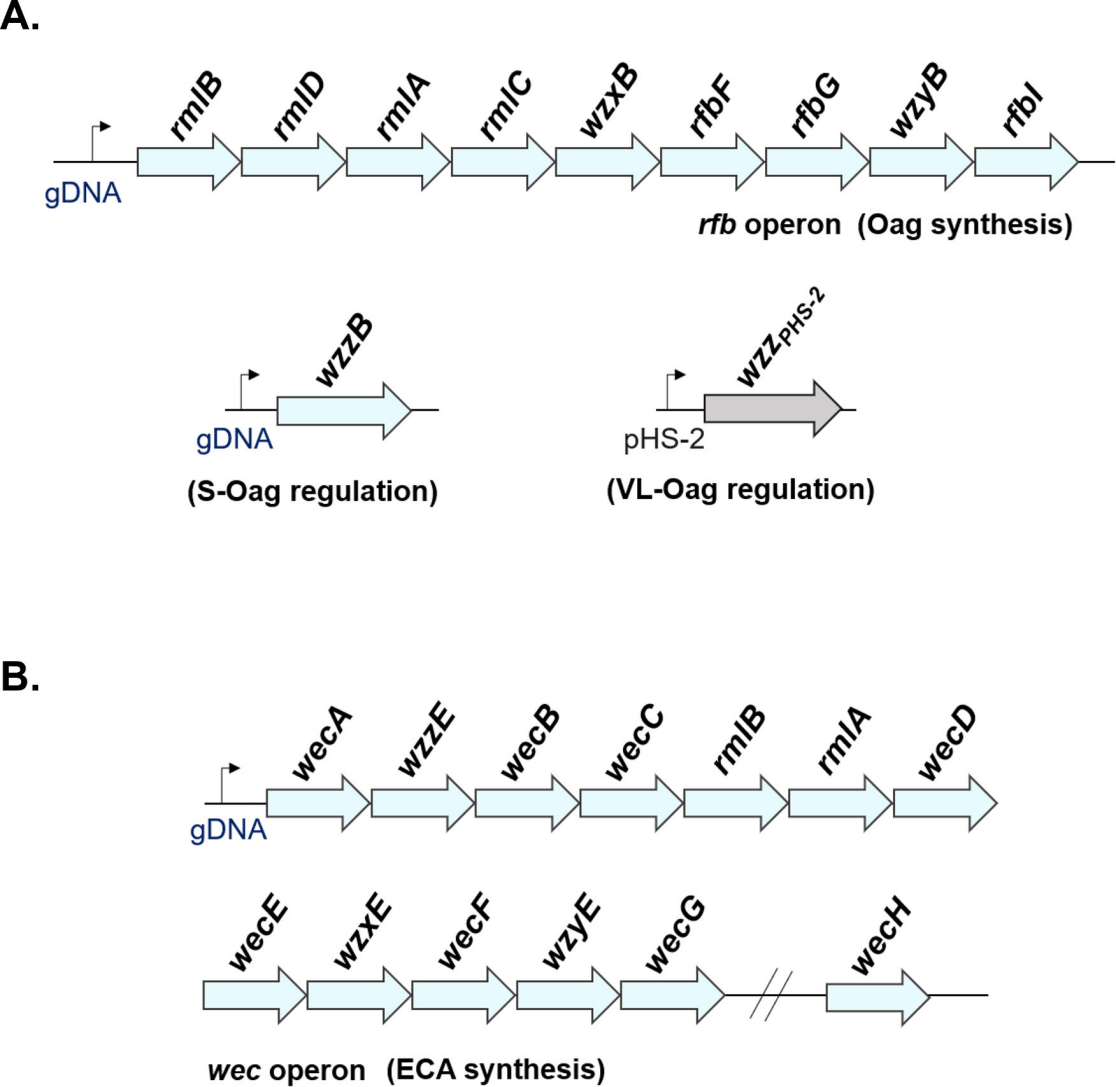
Genetic organization of *S. flexneri wzy* homologs. Schematic illustrating the chromosomal organization of the A. *rfb* operon, *wzzB,* and *wzz_pHS-2_* genes (involved in *S. flexneri* LPS Oag synthesis), and B. the *wec* operon (involved in *S. flexneri* ECA synthesis). Genes are not depicted to scale.

Importantly, this clustering of *wzy* with other genes involved in polysaccharide synthesis and assembly has since been corroborated for a plethora of microbes, albeit the loci differ widely at the bacterial species or genus level. For instance, for *E. coli* and *Salmonella enterica*, LPS Oag *wzy* is usually localized between *gal* and *gnd* genes (67), whereas in most *Yersinia* spp., *wzy* genes are flanked by *hem* and *gsk* (45, 68). In *S. flexneri*, the majority of LPS Oag biosynthetic genes, including *wzyB*, are organized within the chromosomal *rfb* operon (Fig. 3A), with the exception of *wzz_SF_* and *wzz_pHS-2_*, which are independently found in adjacent chromosomal and plasmid-borne (pHS-2) loci, respectively (11, 69). Similarly, for ECA synthesis, *wzyE* is characteristically organized within the chromosomal *wec* operon (Fig. 3B), which is highly conserved across all *Enterobacteriaceae* (12, 70, 71). Exceptions to this chromosomal *wzy* clustering have been reported; for instance, *S. enterica* group B1 LT2 *wzy* is located outside the LPS Oag gene cluster (64, 72), and *Shigella sonnei* possesses its entire Oag gene cluster on an ectopic virulence plasmid following its acquisition from *Plesiomonas shigelloides* (73).

Notably, *wzy* genes differ greatly among bacterial species, exhibiting considerably low amino acid sequence identity (6, 28). Subsequently, the identification of Wzy homologs and orthologs in nature has presented a significant challenge, as canonical sequence-, peptide-, and structure-based approaches have proven insufficiently informative. One clue to identifying putative *wzy* genes is the presence of multiple rare codons at the 5′ translation initiation region of the open reading frame (ORF), which have been reported to serve a regulatory role in polymerase expression and contribute to the remarkably low basal expression of these proteins (74–77). *wzy* ORFs also contain a characteristically lower proportion of GC content compared to the chromosomal average, another factor believed to contribute to their low cellular expression (74). Recent improvements in whole-genome sequencing technology have facilitated pangenome analyses of Oag gene islands (78). This analysis comprehensively defined the genetic basis for Oag variation in *Salmonella* spp., identifying a strong correlation between *wzy* diversity and Oag specificity and observed multiple examples in which Oag processing genes themselves readily change in response to changes in the polysaccharide substrate (42, 78).

### Wzy substrate specificity

Despite multiple suggestions and reports identifying a correlation between Wzy diversity with substrate specificity (3, 8, 45, 79), the intricacies of this specificity between polymerase and cognate substrate remain poorly characterized. Currently available data stem principally from cross-complementation studies of LPS Oag *wzy* polymerases in different bacterial strains and species. In 2012, Kim et al. unsuccessfully complemented a *Francisella tularensis wzy* mutant using *S. flexneri* (serotype 2 a) Wzy expressed *in trans*; an unsurprising result given the distinct contrasts in sugar residue composition (in particular the rare sugar moieties in *F. tularensis* LPS Oag RUs) and different oligosaccharide side-chain modifications (80–82). The inability of Wzy Oag polymerases to successfully cross-complement was then also observed across bacteria of the same species. Genetic mixing experiments in an *S. enterica* model demonstrated that Wzy polymerases from serotypes A, B1, and D1, which retain identical medial LPS Oag trisaccharide RU composition, could result in successful cross-complementation of respective *wzy* mutants (66, 83, 84), with an expected α1→6 linkage produced between RUs during polymerization (72, 75). Interestingly, these Wzy homologs could not successfully complement a serotype C2 *wzy* mutant, which instead produces an Oag polymer composed of α1→4 linked tetrasaccharide RUs containing different glycan substituents (72, 85). This failure to cross-complement across serogroups not only corroborated the high degree of Wzy adaptation to the length of its cognate substrate (Oag RU) but also underpinned the polymerase’s specificity to the composition of the sugar constituents, particularly the sugars at the distal end of the oligosaccharide repeats. This substrate-specificity phenomenon was again recently exemplified by Kasimova et al. (86) in an independent system, following the identification of a secondary *wzy* in *Acinetobacter baumannii*, responsible for synthesis of the capsular polysaccharide, with high specificity to its RUs containing a novel terminal sugar moiety (86–88).

Interestingly, analyses of the Oag biosynthetic system in *P. aeruginosa* demonstrated that while Wzy polymerases have a high specificity for different cognate Oag substrates, identical polymerases can also produce distinct LPS Oag polymers. Notably, the Wzy polymerases expressed by *P. aeruginosa* serotypes O5 and O18/O20 are identical in peptide identity and polymerize LPS Oag trisaccharide RUs composed of identical medial sugar residues, with the exception of the distal moiety which sees the incorporation of epimeric residues D-mannose and L-gulose, respectively. A similar phenomenon is also observed for *P. aeruginosa* serotypes O2 and O16. It has been speculated that these phenotypes may stem from the Wzy polymerases correctly orienting the conformation of the hydroxyl group of the nascent “donor” Oag RU with that of the growing “receiver” polysaccharide during polymerization (8, 23, 36, 89); however, further investigation into the particulars of this molecular mechanism is required. Collectively, Wzy polymerases exhibit a high degree of substrate specificity, which facilitates the synthesis of a wide repertoire of polysaccharides across bacteria.

### Wzy topology

Over the last 30 years, Wzy homologs and orthologs have been investigated in a variety of bacterial models including foremost *P. aeruginosa* serotypes O5 and O3, *F. tularensis*, *E. coli* O86, *Salmonella* spp., and *S. flexneri* (6, 28, 39, 74, 90). In addition to their detection, the low peptide identity shared by Wzy proteins has proven challenging for the isolation of conserved Wzy regions pivotal for their structure and function. Nonetheless, the topology of Wzy proteins responsible for LPS Oag synthesis has been mapped using PhoA::LacZ reporter fusions in *P. aeruginosa* and *S. flexneri* models (6, 74). These studies revealed that the proteins are characteristically comprised of 10–14 α-helical TM segments, thus forming multiple cytoplasmic loops (CLs) and periplasmic loops (PLs) (6, 33, 38). The study focusing on *P. aeruginosa* Wzy (Wzy_PA_) also provided the first insights into the putative catalytic mechanism of the polymerase family. Wzy_PA_ possesses 14 TM domains and 2 large PLs, both of which contain an RX_10_G catalytic motif. Analysis of these two PL “arms” elucidated that, despite a high degree of peptide sequence conservation, they differ drastically in isoelectric point (pI), with overall net positive (pI 8.59) and negative (pI 5.49) charges, respectively (36). Based on these collective observations, Islam and Lam (36) founded the “catch-and-release” model, which describes that the different PL pIs enable them to serve as “capture” and “retention” arms, respectively, mediating transient interaction with the growing polysaccharide chain during polymerization. Subsequent characterization of other Wzy homologs in distinct Gram-negative backgrounds, such as *E. coli* O86, revealed that Wzy_EC:O86_ also possesses two large catalytic PLs, PL3 and PL4, with pI values compared to those of Wzy_PA_ PL3 and PL5, respectively (39). Hence, at the physiological pH, it is believed that Wzy_EC:O86_ also follows a similar catalytic mechanism to the catch-and-release (38). In subsequent analyses, Collins et al. (91) then speculated that the Wzy PL may also interact with the undecaprenyl phosphate group of Oag RUs during polymerization (91). Albeit the nature of the interaction has yet to be defined, this periplasmic region of Wzy polymerases is highly hydrophobic as a result of its abundance of α-helical structures and, notably, was found to exhibit a high degree of *in silico* sequence homology to the β-barrel region of acyl-CoA dehydrogenase, known to function as a fatty acid binding site (75, 92). These speculations were fueled by mutational studies that experimentally demonstrated the importance of the Wzy PL in polymerase function (36, 38).

**Fig 4:**
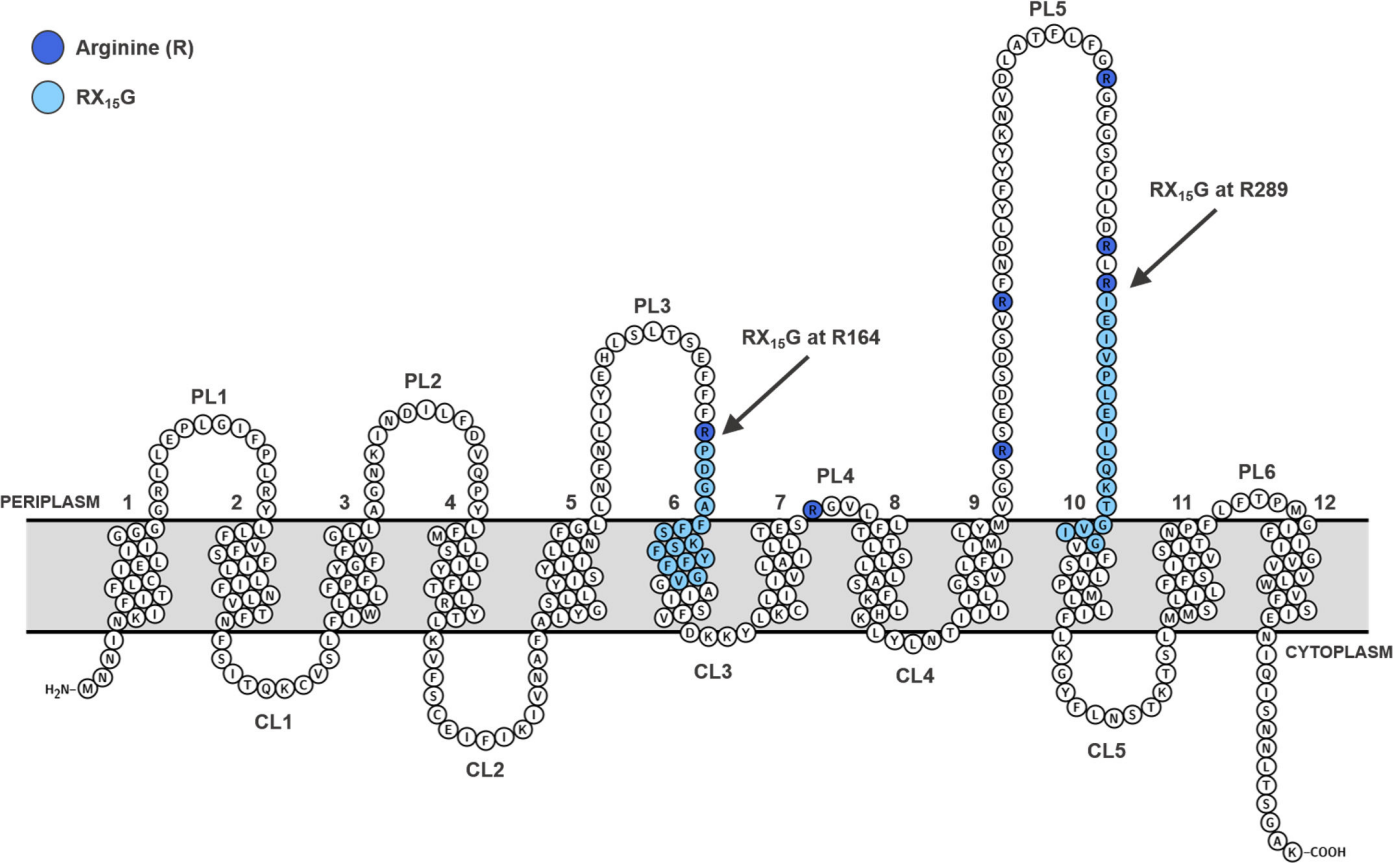
An overview of *S. flexneri* Wzy topology. Wzy_SF_ is a polytopic, integral IM protein, which also contains two large PLs, PL3 and PL5, both containing a functional RX_15_G motif (light blue). These motifs start at residues R164 and R289 (dark blue), respectively, and have been shown to be involved in Wzy function. Wzy_SF_ also contains additional arginine residues (dark blue) situated proximally between PLs 3 and 5, which, similar to the catalytic PLs, also result in a partial or complete loss of Oag polymerization activity if mutated. Figure adapted from (37).

Similarly, the *S. flexneri* Oag polymerase, Wzy_SF_, is also a hydrophobic integral, polytopic IM protein with 12 TM domains, 5 CLs, and 6 PLs (Fig. 4) (38, 74). The differences in the number of TM segments and the size of PLs between Wzy_SF_ and Wzy_PA_ have been attributed to reflect the differences in substrate specificities across the two strains, given that *S. flexneri* serotypes polymerize tetrasaccharide Oag RUs commencing with GlcNAc, whereas *P. aeruginosa* instead utilizes trisaccharide RUs beginning with FucNAc. Furthermore, the S. *flexneri* Oag RUs do not carry a charge (38), whereas *P. aeruginosa* instead polymerizes negatively charged repeats due to their aforementioned incorporation of uronic acid residues in the RUs (22). Similar to Wzy_PA_, Wzy_SF_ also contains two large PLs, 3 and 5, which contain a similar functional motif, RX_15_G, starting at residues R164 and R289, respectively (Fig. 4). However, antithetical to Wzy_PA_ and Wzy_EC_, at physiological pH, the Wzy_SF_ catalytic arms exhibit pI values of PL3 4.65 and 5.09, respectively, indicating that both possess a net negative charge. Collectively, this suggests that a modified version of the catch-and-release mechanism may be operating for Wzx/Wzy-dependent Oag biosynthesis in *S. flexneri*, and perhaps other *Enterobacteriaceae*, which has yet to be defined (38).

### Wzy structure and interactions

Beyond topology mapping and mutant analyses, the intrinsic low basal cellular expression and high hydrophobicity of Wzy proteins have posed challenges in the overexpression and purification of any polymerase in quantities that would facilitate either X-ray crystallography or, more appropriately, cryo-electron microscopy (cryo-EM). Thus, to date, no structure has yet been experimentally defined for any ortholog. This gap in the literature is perpetuated by the many methods routinely implemented to examine the structure and folding of soluble proteins (93), posing a significant challenge when applied to polytopic, integral proteins, such as Wzy, which instead necessitate specific purification requirements and exhibit greater protein instability (94, 95).

The first insights into Wzy conformation were first detected by Daniels et al. (74), who reported that truncated Wzy_SF_ (fused to PhoA) underwent dimerization, forming complexes able to withstand even under the reducing and denaturing conditions of SDS–PAGE (74). These findings were further corroborated by both Zhao et al. in 2014, (39) and Nath and Morona in 2015, who illustrated the same dimerization phenotype in purified Wzy_EC:O86_ (39) and Wzy_SF_ (37), respectively. Subsequently, more recent studies, however, have failed to detect the same dimerization phenomenon (28, 29, 96). Preliminary studies interrogating Wzy interactions demonstrated the formation of a stable complex between Wzy–Wzz *in vivo*, using chemical crosslinking but were unable to identify the direct binding of polymerase to substrate (37, 96). Importantly, the first detection of Wzy_SF_ natively in complex with both its Wzz polysaccharide co-polymerase and Und-PP linked Oag RUs was recently observed by our group in the absence of chemical crosslinking (29), experimentally confirming all three entities naturally interact in complex.

In a related, parallel study, using a novel approach to interrogate the conformation of Wzy within its niche membrane environment, our group also demonstrated the high dynamicity of the LPS Oag polymerase in an *S. flexneri* model, whose conformation is significantly influenced by environmental flux, such as changes in temperature and interaction with its polysaccharide co-polymerase proteins (Wzz), but, interestingly, not the substrate itself (28). These analyses implemented the use of the thiol-reactive probe maleimide-polyethylene glycol to interrogate native Wzy_SF_ conformation *in situ*, given the importance of the adjacent lipid/phospholipid landscape in maintaining the wild-type conformation of membrane proteins (97–101). Interestingly, this work also demonstrated that Wzy_SF_ undergoes distinct conformational changes upon interacting with either the S-Oag or VL-Oag LPS polysaccharide co-polymerases, WzzB_SF_ and Wzz_pHS-2_, respectively. In combination with mutagenesis analyses and reciprocal, native protein co-purification, Wzz_SF_ and Wzz_pHS-2_ were confirmed to compete for discrete, unique Wzy binding sites (28, 29), experimentally addressing previous hypotheses that the two polysaccharide co-polymerases may instead be in direct competition for the same polymerase binding domain (47). Collectively, our recent studies suggested a novel biosynthetic mechanism for VL-Oag LPS, in which the polymerization of this Oag modal length occurs via the repeat addition of nascent Und-PP-linked Oag chains (~30 RUs) as nucleophilic intermediates for chain extension, rather than individual RUs. This process may result in a more entropically favorable synthesis process (29). In the future, these experimentally defined interactions should be further investigated using cutting-edge techniques, such as cryo-EM and nuclear magnetic resonance (NMR).

### Wzz polysaccharide co-polymerases

Wzz proteins are grouped into three classes based on their association with the Wzx/Wzy-dependent pathway, the chemical nature of the polysaccharide they produce, and the presence/absence of additional cytoplasmic domains. Specifically, the Wzz proteins responsible for modulating LPS Oag or ECA lengths are a part of polysaccharide co-polymerase group 1 (PCP1) proteins, which are further subdivided into two categories: (i) PCP-1a, which includes WzzB and WzzE, and (ii) PCP-1b, which includes the polysaccharide co-polymerases that regulate the production of longer modal lengths, such as Wzz_pHS-2_ and FepE (48, 49, 102). In contrast to Wzy proteins, multiple studies have successfully resolved the structure of Wzz homologs/orthologs. PCP1 proteins demonstrate low peptide sequence identity but maintain a remarkably high degree of structural conservation. Wzz monomers are typically ~40 kDa and are composed of two superhelical, right-handed, trans-IM domains located at the protein termini, which are separated by a large soluble, periplasmic domain (91). This periplasmic domain consists of a predicted coiled-coil region, in addition to functional, conserved sequence motifs, in particular proline- and glycine-rich segments, found proximal and within the second TM (TM2) segment (103). Notably, these TM2 motifs have shown to be highly conserved across an array of Wzz homologs/orthologs, including WzzB_SF_ and Wzz_pHS-2_ (104).

Following the resolution of the *S. enterica* Typhimurium WzzB_ST_ periplasmic domains via X-ray crystallography, the protein was found to preferentially exist as an oligomer and occupy variable stable states, primarily trimers to octamers, but in some instances even larger protomer complexes containing 10 to 15 units (105, 106). Together, the oligomerized Wzz monomers form a distinct bell- or dome-shaped quaternary structure (91, 105–107). Subsequent analyses of the reconstituted full-length protein identified that the most common, entropically favorable formation for WzzB_ST_ is either hexameric (108) or octameric (105). More recently, the full-length structure of the WzzB_ST_ oligomeric complex was resolved using Cryo-EM, revealing that the monomers oligomerize through specific interactions mediated by the cytoplasmic domains (91). Additionally, the TM2 region of Wzz has been implicated in facilitating interaction with Wzy (109). Collectively, the data from these studies were recently corroborated by the high-resolution Cryo-EM structure solved for *Pectobacterium atrosepticum* WzzE, which elucidated that Wzz proteins exist as an octameric complex formed by four dimers arranged in C4 symmetry (110). This arrangement was also independently supported by the *E. coli* WzzE cryo-EM structure, which demonstrated that the Wzz octamers are arranged with alternating up-down conformation of the L4 loops and flanking helices, creating a negatively charged luminal binding surface in which the polysaccharide chain is potentially elongated (111). Experimentally, mutation of Wzz TMs yields phenotypically inactive polysaccharide co-polymerase proteins, which subsequently culminates in a loss of appropriate Oag modal chain length regulation (48, 91).

### Wzy–Wzz interplay

**Fig 5:**
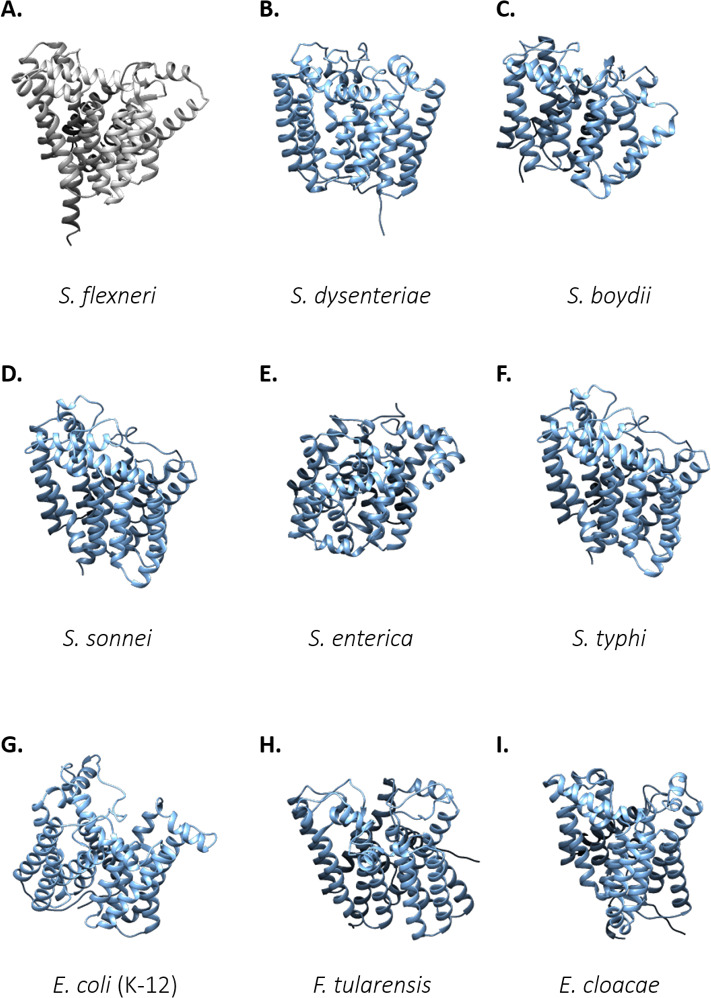
Structural comparison of Wzy orthologs. Structural comparison of Wzy Oag polymerases from distinct bacterial species and serotypes, including (A) *S. flexneri*, (B) *S. dysenteriae*, (C) *S. boydii*, (D) *S. sonnei*, (E) *S. enterica*, (F) *S. typhi*, (G) *E. coli* (K-12), (H) *F. tularensis*, and (I) *E. cloacae*. Despite low amino acid identity, Wzy orthologs exhibit a high degree of structural similarity, with multiple polytopic, hydrophobic, alpha-helical domains arranged in a globular-like three-dimensional, tertiary conformation. Structures were retrieved from the AlphaFold2 server and subsequently analyzed using the USFC Chimera software.

**Fig 6:**
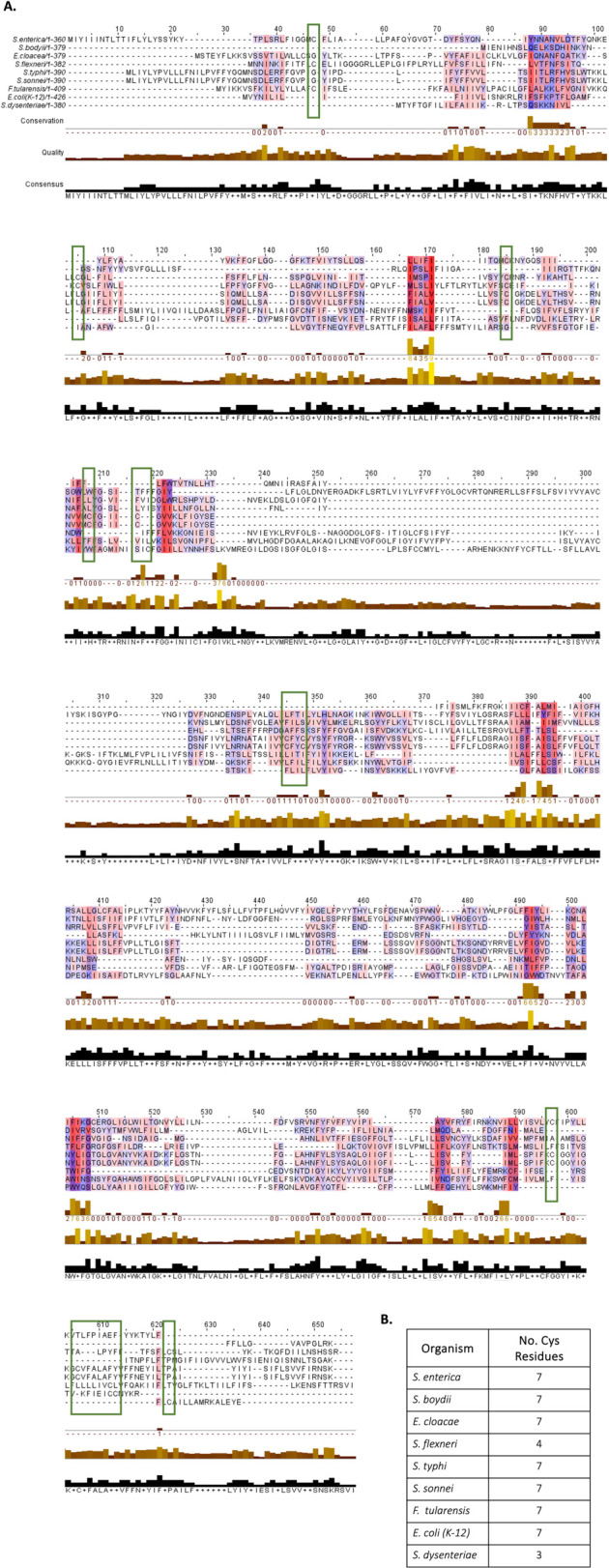
Multiple sequence alignment of Wzy orthologs. (A). Multiple sequence alignment (MSA) of Wzy Oag polymerases from distinct bacterial species and serotypes, including *S. enterica*, *S. boydii*, *E. cloacae*, *S. flexneri*, *S. typhi*, *S. sonnei*, *F. tularensis*, *E. coli* (K-12), and *S. dysenteriae*. Wzy orthologs exhibit low overall amino acid identity (<19.5%) (71) but nonetheless maintain a high degree of cysteine (C) conservation, with residues closely aligning across the polymerases (green boxes). Moieties highlighted red/blue evidence regions of high hydrophobicity (with a 15% conservation threshold). The MSA was generated in Jalview (116) using a Clustal OWS alignment of protein sequences retrieved from UniProt. (B) Summary of Wzy cysteine residues across the aforementioned orthologs.

Over the past decades, a variety of studies have investigated the nature of the Wzy–Wzz interplay; however, given the absence of Wzy structural information, it has proven difficult to gain insight into the interaction kinetics of these proteins. Recently, our interrogation of Wzy–Wzz interactions in an *S. flexneri* model underpinned the importance of Wzy cysteine residues in polymerase conformation and, consequently, in LPS Oag synthesis (28). Wzy cysteine moieties are based principally at the NTD of the polymerase and, notably, were observed to be both spatially close and favorably oriented toward a short stretch of amino acids (351–357) situated at the Wzy CTD (29). Together, these cysteine residues and amino acids 351–357 were found to form an intramolecular NTD-CTD Wzy_SF_ pocket which specifically modulates Wzz_pHS-2_ interaction. Disruption of the Wzy_SF_ cysteine moieties (29) or the CTD aa (351–357) (96) results in impaired interaction with the VL-Oag chain length regulator but elicits no effect on Wzy interaction with the S-Oag co-polymerase, WzzB_SF_. Beyond *S. flexneri*, Wzy polymerases contain a high abundance of cysteine moieties. Via *in silico* sequence and protein structure analyses, Wzy orthologs from a variety of distinct bacterial backgrounds, including *E. coli*, *S. flexneri*, *P. aeruginosa*, *F. tularensis*, *S. enterica*, and *Enterobacter cloacae,* were all found to display similar spatial conformations, despite their low overall amino acid identity (<19.5%) (Fig. 5). Interestingly, even with this poor sequence homology, Oag polymerases do maintain a certain degree of cysteine conservation (Fig. 6A and B). This is particularly noteworthy given that cysteines are the least abundant amino acid found in α-helical structures (112), thus suggesting a real requirement for these proteins to have maintained these residues despite evolving independently. Recent literature has also demonstrated that conservation, specifically of cysteine moieties, most frequently occurs within a protein’s functional domain (binding, catalytic, or regulatory) (113). Beyond *S. flexneri*, an array of bacterial pathogens also rely on bimodal LPS Oag expression as a key virulence factor, including *E. coli* and *S. typhimurium*, through the expression of the Wzz_pHS-2_ homolog, FepE (30, 114, 115). Given (i) the conservation in cysteine residues (Fig. 5), (ii) the similarities in Wzy tertiary structure (despite low amino acid identity) (Fig. 5), and (iii) the high structural conservation across polysaccharide co-polymerase proteins (including Wzz_pHS-2_ and FepE), it is highly likely that a similar NTD-CTD domain exists in orthologous Wzy proteins, which can mediate interaction between the polymerase and its respective VL-Oag chain length regulator.

The identification of this interaction site between a Wzy polymerase and its corresponding VL-Oag chain length regulator is an important finding, as it presents a putative candidate for drug/inhibitor discovery. VL-Oag LPS has been shown to be a critical molecule for pathogens like *S. flexneri* to maintain optimum virulence, as it provides protection against antimicrobials and antibiotics (2, 11, 31) while also serving a key role in the bacterium’s adaptation to its niche host environment *milieu* (10). The isolation of suitable protein inhibitors is challenging in the absence of starting hits or leads. Fortunately, a biologically relevant molecule may already exist for the inhibition of Wzy–Wzz_pHS-2_. The inhibitor of α-polymerase (IAP) is a small peptide (31 aa) expressed by the temperate bacteriophage D3 (117). In a study by Taylor et al. (117) using a *P. aeruginosa* model, IAP was found to successfully mimic the second TM region of Wzz co-polymerases, which is the region necessary for Wzz interaction with Wzy (103, 109). Thus, this small peptide can act as a competitive inhibitor, preferentially binding to Wzy in place of Wzz, and may serve as an important starting point to ultimately develop an inhibitor for VL-Oag LPS synthesis in *S. flexneri* or, ideally, more broadly across enterobacterial pathogens that express dual Oag chain length regulators.

### Wzy–Wzz–Oag interplay during polymerization

In addition to interrogating solely protein–protein interactions, structural analyses have also attempted to understand how Wzy–Wzz interact in complex with the Oag substrate. In 2017, Collins et al. hypothesized that during Oag polymerization, the growing nascent chain either associates with the external Wzz surface or is physically sequestered into the dome-shaped oligomer. Polymerization is proposed to terminate once the binding capacity of the polysaccharide co-polymerase is reached (91). Recently, Weckener et al. (110) have invalidated the former hypothesis, demonstrating that the outer surface of *P. atrosepticum* WzzE does not act in the ruler mechanism for ECA synthesis in this pathway. Using a series of structural and biochemical approaches, Weckener and colleagues instead revealed that both the polymerase and nascent polysaccharide are situated within the Wzz lumen during polymerization (110). These findings are supported by data interrogating FepE from *E. coli* and WzzB from *S. flexneri*, where mutations within the lumen affected Oag chain length regulation (115). Overall, current data support a model whereby the Oag polysaccharide grows until it has filled the Wzz periplasmic lumen, and upon reaching maximum capacity, this process prompts the destabilization of the Wzz octamer and the release of the polysaccharide chain, thus terminating Oag biosynthesis (110).

### Conclusion

Considerable progress has been made in understanding the mechanism of polysaccharide biosynthesis via the Wzx/Wzy-dependent pathway since its initial conception decades ago. As detailed in this review, many recent studies investigating individual pathway constituents, in particular Wzy and Wzz, and their interactions have provided significant novel knowledge furthering our understanding of this important, widespread system for bacterial glycan biosynthesis. Collectively, data from these studies by multiple laboratories worldwide have also paved the direction for future work, underpinning the importance of obtaining full-length structural data for Wzx/Wzy/Wzz, ideally in native complex with the polysaccharide substrate, using high-resolution X-ray crystallography, cryo-EM, or NMR techniques.

The integration of complementary data from an array of fields, including bacterial genetics, biochemistry, bioinformatics, and molecular microbiology, will be required in order to successfully describe the nature of the stoichiometric interplay between the different assembly pathway constituents during polysaccharide polymerization. Nonetheless, the ongoing investigation into this pathway and its constituents continues to yield novel and exciting insights. As summarized in this review, these insights are stimulating the detailed characterization of the most common biosynthetic pathways for bacterial SPs, which remain one of the most important virulence factors for countless debilitating and lethal pathogens.
